# Serum IgG Antibody Levels to Periodontal Microbiota Are Associated with Incident Alzheimer Disease

**DOI:** 10.1371/journal.pone.0114959

**Published:** 2014-12-18

**Authors:** James M. Noble, Nikolaos Scarmeas, Romanita S. Celenti, Mitchell S. V. Elkind, Clinton B. Wright, Nicole Schupf, Panos N. Papapanou

**Affiliations:** 1 Taub Institute for Alzheimer Disease and the Aging Brain, Columbia University, New York, New York, United States of America; 2 Department of Neurology, College of Physicians and Surgeons, Columbia University, New York, New York, United States of America; 3 Department of Social Medicine, Psychiatry, and Neurology, National and Kapodistrian University of Athens, Athens, Greece; 4 Division of Periodontics, Section of Oral and Diagnostic Sciences, Columbia University College of Dental Medicine, New York, New York, United States of America; 5 Department of Epidemiology, Mailman School of Public Health, Columbia University, New York, New York, United States of America; 6 Evelyn F. McKnight Brain Institute, Departments of Neurology and Epidemiology & Public Health Sciences, and the Neuroscience Program, Leonard M. Miller School of Medicine, University of Miami, Miami, Florida, United States of America; Boston University, United States of America

## Abstract

**Background:**

Periodontitis and Alzheimer disease (AD) are associated with systemic inflammation. This research studied serum IgG to periodontal microbiota as possible predictors of incident AD.

**Methods:**

Using a case-cohort study design, 219 subjects (110 incident AD cases and 109 controls without incident cognitive impairment at last follow-up), matched on race-ethnicity, were drawn from the Washington Heights-Inwood Columbia Aging Project (WHICAP), a cohort of longitudinally followed northern Manhattan residents aged >65 years. Mean follow-up was five years (SD 2.6). In baseline sera, serum IgG levels were determined for bacteria known to be positively or negatively associated with periodontitis (*Porphyromonas gingivalis*, *Tannerella forsythia, Actinobacillus actinomycetemcomitans* Y4, *Treponema denticola, Campylobacter rectus, Eubacterium nodatum,* and *Actinomyces naeslundii* genospecies-2). In all analyses, we used antibody threshold levels shown to correlate with presence of moderate-severe periodontitis.

**Results:**

Mean age was 72 years (SD 6.9) for controls, and 79 years (SD 4.6) for cases (p<0.001). Non-Hispanic Whites comprised 26%, non-Hispanic Blacks 27%, and Hispanics 48% of the sample. In a model adjusting for baseline age, sex, education, diabetes mellitus, hypertension, smoking, prior history of stroke, and apolipoprotein E genotype, high anti-*A. naeslundii* titer (>640 ng/ml, present in 10% of subjects) was associated with increased risk of AD (HR = 2.0, 95%CI: 1.1–3.8). This association was stronger after adjusting for other significant titers (HR = 3.1, 95%CI: 1.5–6.4). In this model, high anti-*E. nodatum* IgG (>1755 ng/ml; 19% of subjects) was associated with lower risk of AD (HR = 0.5, 95%CI: 0.2–0.9).

**Conclusions:**

Serum IgG levels to common periodontal microbiota are associated with risk for developing incident AD.

## Introduction

Poor oral health, including caries, periodontal disease, and edentulism are highly prevalent globally, particularly in the elderly. [1] Caries and periodontitis share common risk factors, including infectious etiologies, and are main contributors to tooth loss in elderly populations. [2] Periodontitis prevalence estimates among adults vary widely (20%–>80%), [1], [3] and this variability is partly attributed to varying disease definitions. [4]

Periodontitis is a chronic inflammatory disease, initiated by a bacterial biofilm adhering to the tooth surfaces adjacent to the gingiva. [5], [6] Oral colonization by periodontal bacterial pathogens^6^ is ubiquitous in older adults, [7] with a significant proportion of the population exposed by adolescence. [8] A systemic host response to periodontal microbiota is manifested through the presence of antibacterial antibodies in the serum [9] as well as by elevated inflammatory cytokines. [10]

Epidemiological evidence supports an association between the level of serum antibodies to periodontal pathogens and stroke, [11]–[13] and accelerated aortic atherogenesis. [14] High levels of colonization by specific periodontal pathogens are associated with increased carotid artery intimal-medial thickness. [15] Risk factors for stroke and dementia have a similar systemic inflammatory profile to periodontitis [16] and suggest a final common pathway of atherogenesis related to systemic inflammation. [16]

Poor dental status, a late-life marker of the cumulative effects of oral inflammatory pathologic conditions including periodontitis, is associated with prevalent cognitive impairment and incident dementia. [17] We previously identified a cross-sectional association between high serum IgG to a common periodontal pathogen, *Porphyromonas gingivalis,* and poor cognitive test performance among people aged >60 years in the Third National Health and Nutrition Examination Survey (NHANES-III). [18] Another group reported that TNF-α levels in combination with three periodontal IgG titers could discriminate between patients with Alzheimer disease (AD) and cognitively normal individuals. [19] Studies exploring associations of serologic markers of periodontitis and cognition have been limited to cross-sectional analyses and neuropsychological measurements. [17] A growing body of evidence associating periodontitis with stroke [20] and cognitive impairment [17] justifies further investigation of a possible association between poor oral health and incident dementia.

In this study, pre-morbid levels of serum IgG antibodies to selected periodontal microbiota were explored for possible association with risk for incident AD. We hypothesized that serologic markers of periodontitis can serve as predictors of incident cognitive impairment among older adults, and tested this hypothesis in a case-cohort study of the Washington Heights Inwood Columbia Aging Project (WHICAP).

## Methods

### Data source

WHICAP has longitudinally followed a multiethnic elderly community population in northern Manhattan with serial neuropsychological assessments repeated every 18–24 months. Study design, criteria for diagnosis of AD, and vascular risk factor definitions have been described elsewhere. [21], [22] The subset included in the present analyses consisted of WHICAP participants enrolled in 1999–2000 with no prevalent cognitive impairment or dementia at the time of phlebotomy and ≥1 follow-up visits thereafter. Archived sera were available from phlebotomized subjects at the first follow-up visit which occurred in 2001 for most participants; presence of individual vascular risk factors was determined at the first visit.

### Study design

The present study has a case-cohort design, comparing individuals with incident probable Alzheimer disease with controls without incident dementia through the last available follow-up, matched by race-ethnicity. This design is advantageous for the purposes of an exploratory study because of its cost-efficiency, [23] but may be prone to sampling bias, and thus warrants conservative statistical analyses. [23], [24]

### Determination of cases and controls

Individuals to be included in this study were selected in October 2010. Cases were identified as persons without prevalent AD at the time of phlebotomy who were subsequently determined clinically to have AD during follow-up. [21] Controls were identified based on absence of clinical dementia at the time of the last available follow-up. Cases and controls were proportionately matched based on race-ethnicity, given the known impact of race-ethnicity on antibody titers, [25] rates of dementia, [26] and cognitive decline. [27] Control participants were stratified by race-ethnicity and then randomly selected for inclusion.

Thus, the sample included 135 incident AD cases and 134 controls, of whom 110 cases and 109 controls had available serum. Controls comprised approximately 10% of the overall control sample; for these analyses, the sampling fraction was equal to 1 for cases and 1/10 for controls.

### Assessment of serum IgG antibody levels

Serum IgG antibody levels to periodontal bacteria were determined by means of checkerboard immunoblotting [28] as previously described. [29] Assessments were carried out for bacteria with known positive or negative associations with clinical periodontitis: [9] *Porphyromonas gingivalis* (strain FDC381), *Tannerella forsythia* (ATCC43037), *Actinobacillus actinomycetemcomitans* (FDCY4), *Treponema denticola* (OMGS3271), *Campylobacter rectus* (ATCC33238), *Eubacterium nodatum* (OMGS3356), and *Actinomyces naeslundii* genospecies 2 (ATCC15987).

### Periodontal antibody threshold levels

Antibody threshold levels were used as a proxy for exposure to common periodontal pathogens, based on the strong associations with clinical periodontitis identified by our group in NHANES-III. [9] At-risk individuals were defined using two *a priori* approaches: a) those exceeding single antibody thresholds distinguishing clinical periodontitis from healthy periodontal conditions [9] and b) those in the highest serum antibody level quintile. In NHANES-III, the clinical threshold values corresponded to the 90^th^ percentile titer of individuals free of periodontitis according to the definitions by the Centers for Disease Control and Prevention/American Academy of Periodontology (CDC/AAP). [30] Because two sets of clinical thresholds were identified for the two NHANES-III phases, the lower of the two were used in the present analyses.

### Statistical analyses

Descriptive analyses used t-tests for comparing means of continuous variables, and χ^2^ tests for comparing categorical variables. Multivariable Cox proportional-hazards regression models were used to examine individual associations between each of the seven antibody thresholds and incident AD. Time-to-event was defined as duration from phlebotomy to time of AD diagnosis. To minimize small-sample bias, conservative methods suited for case-cohort analyses were used, including derivation of an “exact” pseudolikelihood estimator for the point estimate, and robust variance estimation for derivation of the 95% confidence interval for each association. [23], [24], [31] Controls were weighted by the inverse of the sampling fraction. The strength of the association between high antibody titers and dementia status was explored before and after adjusting for selected covariates, including age at enrollment, sex and education, as well as known vascular risk factors including hypertension, diabetes mellitus (DM), hyperlipidemia, stroke history, heart disease, tobacco abuse, and APOE e4 carrier status. Based on power calculations, 100 matched pairs afforded sufficient power to identify ≥38.5 ng/ml difference in mean antibody levels between AD and controls. All analyses were performed on an anonymized and de-identified data set using SAS v. 9.3. The Institutional Review Board of Columbia University Medical Center approved this proposal.

## Results

Cases were older at enrollment, had shorter follow-up time (person-years follow-up: cases = 4.4 years [SD = 2.5], controls = 5.5 years [SD = 2.5], p = 0.001), were less educated, and were more likely to have a history of prior stroke (**Table 1**). In comparison to the overall WHICAP cohort, the participants in the case-cohort study were similar in most regards, although differed in race-ethnic composition, education history, and hypertension prevalence. Percentiles of antibody levels, relative to previously published antibody thresholds for clinical periodontitis, [9] are listed in **Table 2**. High antibody levels to *C. rectus* were found in 69% of the participants, followed by 63% to *T. forsythia*, 53% to *T. denticola*, 23% to *P. gingivalis*, 19% to *E. nodatum,* 11% to *A. actinomycetemcomitans* and 10% to *A. naeslundii*. The proportions did not differ between cases and controls (**Table 2**).

**Table 1:** Characteristics of the study cohort.

				Case-Cohort Study		
Characteristic	Case-cohort subjects (N = 219)	Complete WHICAP cohort (N = 1957)	p-value*	Controls (n = 109)	Cases (n = 110)	p-value**
Age at enrollment, years (SD)	75.6 (6.9)	76.2 (6.5)	0.14	72.3 (4.6)	78.9 (7.2)	<0.001
Women, n (%)	148 (67.6)	1150 (66.2)	0.68	73 (67.0)	75 (68.2)	0.85
Hispanic, n (%)	105 (47.9)	575 (33.1)	<0.001	52 (47.7)	53 (48.2)	0.94
Non-Hispanic Black, n (%)	58 (26.5)	570 (32.8)	—	30 (27.5)	28 (25.5)	0.73
Education, years (SD)	9.9 (5.3)	10.7 (4.7)	0.02	11.9 (4.6)	7.8 (5.1)	<0.001
APOE-∈4, n (%)	54 (25.2)	398 (26.8)	0.50	28 (26.4)	26 (24.1)	0.69
Diabetes, n (%)	41 (18.7)	330 (19.2)	0.87	17 (15.6)	24 (21.8)	0.24
Hypertension, n (%)	125 (57.1)	1172 (68.1)	0.001	62 (56.9)	63 (57.3)	0.95
Stroke history, n (%)	20 (9.1)	184 (10.7)	0.48	3 (2.8)	17 (15.5)	0.001
Current Smoker, n (%)	20 (9.6)	151 (9.5)	0.85	12 (11.5)	8 (7.6)	0.34

P-values based on t-test for continuous variables, and on chi-square for categorical variables.

*Case-cohort study v. complete WHICAP cohort.

**Cases v. controls within the case-cohort study.

**Table 2:** Distribution of serum IgG antibody levels to selected periodontal species.

Periodontal pathogen	Study Subjects IgG levels (ng/ml) (Total N = 219)								*CDC/AAP Clinical Threshold* *	Study Subjects Exceeding CDC/AAP Threshold* n (%)				
	Percentile													
	20^th^	25^th^	40^th^	50^th^	60^th^	75th	80th	Maximum		Total (N = 219)	Controls (n = 109)	Cases (n = 110)	p-value	
*A naeslundii*	0	0	29.8	97.2	163.0	336.9	380.9	13942.2	*640*	22 (10.0)	10 (9.2)	12 (10.9)		0.67
*E. nodatum*	86.6	122.1	318.4	489.0	627.8	1204.1	1609.6	75725.0	*1755*	42 (19.1)	23 (21.1)	19 (17.3)		0.47
*P. gingivalis*	52.2	83.6	200.3	307.6	402.7	672.1	865.3	15398.8	*713*	50 (22.8)	25 (22.9)	25 (22.7)		0.97
*T. forsythia*	32.3	56.7	140.5	194.2	266.1	409.3	482.7	9475.6	*128*	137 (62.6)	72 (66.1)	65 (59.1)		0.29
*T. denticola*	31.1	51.3	105.6	160.2	225.6	353.1	431.5	8317.6	*136*	116 (53.0)	62 (56.9)	54 (49.1)		0.25
*C. rectus*	38.4	61.2	156.7	269.6	356.8	667.8	829.5	16419.4	*112*	150 (68.5)	78 (71.6)	72 (65.5)		0.33
*A. actinomycetemcomitans*	64.7	89.4	186.4	253.4	336.5	507.4	558.6	9284.9	*730*	25 (11.4)	10 (9.2)	15 (13.6)		0.30

*Thresholds are drawn from a study of NHANES-III subjects [9]; above each individual periodontal pathogen level, the accuracy of a serology-based diagnostic test to detect moderate-severe periodontitis according to the CDC/AAP definition is maximized. [30].

Participants with elevated *A. naeslundii* serum IgG (>640 ng/ml) had a consistently higher risk for incident AD (**Table 3**), ranging from 80%–100%, with similar yet less precise estimates emerging from more conservative statistical analyses. This finding remained robust in the fully adjusted Cox model, which included age, sociodemographic variables, vascular risk factors, APOE status, and histories of DM, stroke, tobacco abuse, and hypertension (hazard ratio (HR) = 2.0, 95% CI: 1.1–3.8). In addition, in a fully adjusted model, high antibody levels to *E. nodatum* (>1755 ng/ml) approached statistical significance with a decreased risk of incident AD (HR = 0.7, 95% CI: 0.4–1.2, p = 0.19). Fully adjusted survival curves are provided for each of these analyses in **S1** **and** **S2 Figures**. Next, when high titers to both *A. naeslundii* and *E. nodatum* were included into similar models (**Table 4**), a greater risk of incident AD was observed in the presence of high *A. naeslundii* levels (HR = 3.1, 95% CI: 1.5–6.4), but a lesser risk in the presence of high *E. nodatum* levels (HR = 0.5, 95% CI: 0.2–0.9) in the fully adjusted model. Neither antibody demonstrated evidence of being an effect modifier of the other in this relationship.

**Table 3:** Regression models: Association of high clinical threshold [9] levels of serum IgG to selected periodontal species and incident AD.

Periodontal pathogen	Cox proportional Hazards Regression Model				Pseudolikelihood estimator with robust variance estimator*			
	Model 1 HR (95% CI)	Model 2 HR (95% CI)	Model 3 HR (95% CI)	Model 4 HR (95% CI)	Model 1 HR (95% CI)	Model 2 HR (95% CI)	Model 3 HR (95% CI)	Model 4 HR (95% CI)
*A. naeslundii*	1.8 (1.0–3.2)	**2.1 (1.1–3.8)**	1.8 (1.0–3.4)	**2.0 (1.1–3.8)**	1.6 (0.9–2.8)	**1.8 (1.0–3.1)**	1.4 (0.8–2.7)	1.6 (0.8–3.2)
*E. nodatum*	1.1 (0.7–1.8)	0.8 (0.5–1.4)	0.8 (0.4–1.3)	0.7 (0.4–1.2)	0.7 (0.5–1.2)	0.7 (0.4–1.2)	0.6 (0.3–1.2)	0.6 (0.3–1.2)
*P. gingivalis*	1.1 (0.7–1.7)	0.9 (0.5–1.4)	0.9 (0.6–1.5)	0.9 (0.6–1.5)	1.0 (0.7–1.4)	0.9 (0.6–1.4)	1.0 (0.6–1.6)	1.0 (0.6–1.6)
*T. forsythia*	0.9 (0.6–1.3)	0.9 (0.6–1.4)	0.9 (0.6–1.4)	0.9 (0.6–1.4)	0.9 (0.7–1.3)	0.9 (0.6–1.3)	0.8 (0.6–1.2)	0.8 (0.6–1.2)
*T. denticola*	1.0 (0.7–1.4)	1.0 (0.7–1.4)	0.9 (0.6–1.4)	0.9 (0.6–1.3)	0.9 (0.7–1.3)	0.9 (0.6–1.3)	0.9 (0.6–1.3)	0.8 (0.6–1.3)
*C. rectus*	1.0 (0.6–1.5)	0.9 (0.6–1.4)	0.7 (0.5–1.1)	0.7 (0.5–1.2)	0.9 (0.7–1.3)	0.9 (0.6–1.3)	0.7 (0.5–1.1)	0.7 (0.5–1.1)
*A. actinomycetemcomitans*	1.3 (0.7–2.3)	0.9 (0.5–1.6)	1.1 (0.6–1.9)	1.1 (0.6–2.0)	0.9 (0.6–1.5)	1.0 (0.6–1.8)	1.1 (0.6–2.0)	1.1 (0.6–2.2)

Model 1: bivariate association with single periodontal antibody.

Model 2: controlled for age at phlebotomy (baseline).

Model 3: Model 2, also controlled for APOE status, gender, and education.

Model 4: Model 3, also controlled for hypertension, smoking, stroke, and diabetes mellitus.

*Models shown using pseudolikelihood estimator, robust variance estimator, and weight of cases (1), controls (10) [most conservative method].

Models highlighted in bold indicate p<0.05. HR: Hazard ratio.

**Table 4:** Regression models: Association of high levels of serum IgG to *A. naeslundii* and *E. nodatum* and incident AD.

Periodontal pathogen	Cox proportional Hazards Regression Model				Pseudolikelihood estimator with robust variance estimator*			
	Using antibody clinical thresholds [9]				Using antibody clinical thresholds [9]			
	Model 1 HR (95% CI)	Model 2 HR (95% CI)	Model 3 HR (95% CI)	Model 4 HR (95% CI)	Model 1 HR (95% CI)	Model 2 HR (95% CI)	Model 3 HR (95% CI)	Model 4 HR (95% CI)
*A. naeslundii*	1.8 (0.9–3.4)	**2.6 (1.3**–**5.1)**	**2.6 (1.3**–**5.3)**	**3.1 (1.5**–**6.4)**	**2.3 (1.2**–**4.1)**	**2.6 (1.4**–**4.7)**	**2.4 (1.2**–**5.2)**	**3.1 (1.4**–**6.5)**
*E. nodatum*	0.9 (0.6–1.7)	0.6 (0.4–1.1)	0.5 (0.6–1.0)	**0.5 (0.2**–**0.9)**	**0.6 (0.3**–**1.0)**	**0.5 (0.3**–**0.9)**	**0.4 (0.2**–**1.0)**	**0.4 (0.2**–**0.9)**
	Using 80^th^ percentiles				Using 80th percentiles			
*A. naeslundii*	1.2 (0.7–2.0)	1.5 (0.9–2.5)	**1.9 (1.1**–**3.5)**	**1.9 (1.0**–**3.5)**	1.5 (0.8–2.7)	1.4 (0.8–2.5)	1.9 (1.0–3.8)	2.0 (1.0–4.0)
*E. nodatum*	1.0 (0.6–1.7)	0.7 (0.4–1.2)	**0.5 (0.3**–**1.0)**	**0.5 (0.2**–**0.9)**	**0.6 (0.3**–**0.9)**	0.6 (0.3–1.1)	**0.4 (0.2**–**0.9)**	**0.4 (0.1**–**0.9)**

Model 1: bivariate association with both antibodies included in the model.

Model 2: controlled for age at phlebotomy (baseline).

Model 3: Model 2, also controlled for APOE status, gender, and education.

Model 4: Model 3, also controlled for hypertension, smoking, stroke, and diabetes.

*Models shown using pseudolikelihood estimator, robust variance estimator, and weight of cases (1), controls (10) [most conservative method].

Models highlighted in bold indicate p<0.05. HR: Hazard ratio.

When considering the highest quintile levels for *A. naeslundii* (>380.9 ng/ml) and *E. nodatum* (>1609.6 ng/ml) individually, no significant association with risk of incident AD was identified in these models (**Table 5**). However, when including both antibody highest quintiles simultaneously, a similar association was found in the fully adjusted models (*A. naeslundii* HR = 1.9, 95% CI 1.0–3.5 and *E. nodatum* HR = 0.5, 95% CI 0.2–0.9), as when using the individual thresholds **(** **Table 4** **)**.

**Table 5:** Regression models: Association of high levels (80^th^ percentile) of serum IgG to selected periodontal species and incident AD.

Periodontal Pathogen	Cox proportional Hazards Regression Model				Pseudolikelihood estimator with robust variance estimator*			
	Model 1 HR (95% CI)	Model 2 HR (95% CI)	Model 3 HR (95% CI)	Model 4 HR (95% CI)	Model 1 HR (95% CI)	Model 2 HR (95% CI)	Model 3 HR (95% CI)	Model 4 HR (95% CI)
*A. naeslundii*	1.2 (0.8–2.0)	1.2 (0.8–2.0)	1.3 (0.8–2.1)	1.3 (0.8–2.1)	1.1 (0.7–1.6)	1.1 (0.7–1.7)	1.0 (0.6–1.8)	1.1 (0.6–1.9)
*E. nodatum*	1.1 (0.7–1.8)	0.8 (0.5–1.3)	0.7 (0.4–1.3)	0.7 (0.4–1.2)	0.7 (0.4–1.1)	0.7 (0.4–1.2)	0.7 (0.4–1.2)	0.6 (0.3–1.1)
*P. gingivalis*	1.0 (0.6–1.6)	0.8 (0.5–1.3)	0.9 (0.5–1.6)	0.9 (0.5–1.6)	0.9 (0.6–1.4)	0.9 (0.6–1.5)	1.1 (0.7–1.8)	1.1 (0.6–1.8)
*T. forsythia*	1.0 (0.6–1.7)	1.0 (0.6–1.6)	1.0 (0.6–1.7)	0.8 (0.4–1.6)	0.9 (0.6–1.3)	0.9 (0.6–1.5)	0.9 (0.5–1.7)	0.8 (0.4–1.6)
*T. denticola*	0.9 (0.5–1.5)	0.9 (0.5–1.5)	1.0 (0.6–1.7)	1.0 (0.6–1.8)	0.8 (0.5–1.3)	0.9 (0.6–1.4)	0.9 (0.6–1.6)	1.0 (0.6–1.9)
*C. rectus*	0.9 (0.6–1.6)	0.8 (0.5–1.3)	0.8 (0.5–1.4)	0.8 (0.5–1.4)	0.9 (0.6–1.4)	0.9 (0.6–1.5)	1.0 (0.6–1.7)	1.0 (0.6–1.7)
*A. actinomycetemcomitans*	1.3 (0.8–2.0)	0.9 (0.6–1.5)	1.0 (0.6–1.6)	1.0 (0.6–1.7)	0.9 (0.6–1.3)	1.0 (0.6–1.6)	1.0 (0.6–1.7)	1.1 (0.6–1.8)

Model 1: bivariate association with single periodontal antibody.

Model 2: controlled for age at phlebotomy (baseline).

Model 3: Model 2, also controlled for APOE status, gender, and education.

Model 4: Model 3, also controlled for hypertension, smoking, stroke, and diabetes.

*Models shown using pseudolikelihood estimator, robust variance estimator, and weight of cases (1), controls (10) [most conservative method]; HR: Hazard ratio.

## Discussion

In this community-based, multiethnic cohort of elders, an increased risk of incident AD was observed among participants with high serum *A. naeslundii* IgG antibody, and this finding was further strengthened when high *E. nodatum* levels were also included in the model. To our knowledge, this is the first longitudinal study to identify an association between these serological markers of periodontal infection and incident AD.

There are several plausible mechanisms relating periodontal infections to impaired cognition: [17] First, periodontitis could affect risk of cognitive impairment on the basis of stroke and accelerated atherogenesis. [32], [33] A second possibility is that periodontitis could be related to cognition via subclinical cerebrovascular damage in those without stroke. Similar to DM, hypertension, and smoking, [34] periodontitis is associated with impaired arterial endothelial function [35] which, in turn, has been associated with cerebral white matter hyperintensities, [36] vascular dementia, and AD. [37], [38] Alternatively, periodontitis could be associated with dementia risk through systemic inflammation, a process that has been hypothesized to directly influence expression of neurodegenerative disorders such as AD. [39] Other clinical measures of oral health including tooth loss are associated with incident dementia and suggest a possible effect modification with education and APOE. [17]

The primary finding of the study is that high serum *A. naeslundii* IgG is associated with increased risk of incident AD. *A. naeslundii* is a gram positive rod associated with early dental plaque formation, [40] gingivitis, [41] and dental caries. [42] The relationship between serologic evidence of *A. naeslundii* host response and general health outcomes has not been explored to the same extent as with established periodontal pathogens such as *P. gingivalis* that has been implicated in atherogenesis. [14], [43] Although high levels of *A. naeslundii* antibodies are associated with periodontal health [9], plaque biofilms include high proportions of *A. naeslundii*. [44] *A. naeslundii*, and specifically the peptidoglycan from its cell wall, likely has a strongly pathogenic role in osteoclast activation and alveolar bone loss similar to gram negative periodontal organisms such as *P. gingivalis,* including elevation of IL-1β, IL-6, and TNF-α mRNA. [45] Multispecies microbial biofilms including *A. naeslundii* can elicit strong expression of several cytokines and chemokines in oral epithelial cells, including IL-1α, IL-6, TGFα, IL-8, fractalkine, and interferon gamma-induced protein (IP-10). [46]

Additionally, high serum *E. nodatum* IgG levels were associated with lower AD risk in the most conservative regression models. *E. nodatum* is a gram positive, anaerobic rod that has been implicated in periodontitis. [47] Although its role in oral disease pathogenesis is uncertain, high levels of *E. nodatum* oral colonization are associated with generalized aggressive periodontitis among younger individuals [48] as well as more broadly, with chronic periodontitis in adults. [49]. However, high serum *E. nodatum* IgG was associated with lower risk for clinical periodontitis in NHANES-III. [9] Taken together, these findings underscore the complex role of host immune response in periodontitis, and possibly AD, and these relationships warrant further exploration.

Our group previously reported that high antibody levels to *P. gingivalis* were cross-sectionally associated with poor cognitive test performance among patients aged >60 years in NHANES-III. [18] In the same cohort, clinical markers of periodontitis (gingival bleeding, loss of periodontal attachment, and tooth loss) were associated with impaired attention among 20–59 year olds, [50] but these same clinical measures of periodontitis were not associated with impaired recall among those aged 70 years and older. [50] The cause for the discrepancy between serological and clinical markers of periodontitis with poor cognition is uncertain but could relate in part to the equivocal relationship between serum antibodies to periodontal microbiota and clinical periodontal status, the imprecise clinical periodontal measurements in NHANES-III, [51], [52] or residual confounding. Cross-sectional analyses do not allow for inferences regarding directionality of the association of cognition and dental health.

The clinical significance of a high antibody response to specific periodontal microbiota is uncertain. As previously demonstrated, [25] antibody responses to periodontal bacteria are influenced by multiple factors beyond the clinical periodontal status, including race/ethnicity, smoking habits and likely also unmeasured health behaviors. Host antibody responses to periodontal bacteria could also reflect a co-infection with other microbiota with shared antigenic epitopes, many of which are not readily cultivable. [53] Serum antibody levels to periodontal pathogens remain relatively stable after periodontal therapy, and are markedly lower in periodontally healthy subjects, suggesting antibody levels signify the cumulative exposure to oral pathogens and can help discriminate between subjects with high and low susceptibility to severe disease. [54]

Interestingly, the observed relationships between high levels of IgG to *A. naeslundii* and *E. nodatum* and incident AD were strengthened when these antibody responses were explored jointly in regression models. Taken together, these findings suggest the host response to these two periodontal species, although primarily targeted towards control of the localized periodontal infection, may nevertheless stimulate a systemic inflammatory cascade, and support a hypothesis of mediation of incident AD risk through systemic host inflammatory responses. Since data on clinical periodontal status and inflammatory markers such as CRP were not available in this study, a potential interaction between clinical periodontitis and systemic antibody responses cannot be determined.

Several additional limitations and potential strengths of the present study are acknowledged. Measurable socioeconomic covariates provide a restricted scope of lifelong socioeconomic status, and could lead, at least in part, to residual confounding. However, the influence of socioeconomic differences was likely diminished by matching cases and controls on race-ethnicity. Additionally, adjusting for chronological age alone might not capture residual confounding due to age-related illnesses that are variably distributed between people of similar ages. Although analyses were limited to the study of antibodies to seven periodontal bacteria, these were preselected on the basis of their documented association with clinical periodontal status [9] and thus facilitated a more hypothesis-driven, rather than exploratory approach. Likewise, we consider it a strength of the study that the used threshold values for high serum high IgG levels were based on findings from an independent cohort which identified associations with clinically assessed periodontitis. [9] No significant associations between individual antibacterial antibody levels and incident AD were identified when using the a priori cutoff of the 80^th^ percentile, which as shown in Table 2 differed by up to 7-fold compared with previously established clinical status-associated thresholds. In some regards, this suggests that clinical thresholds may be more important to consider than more conventional quintile or quartile cut-off points. We did not pursue analyses using a sum of high antibody thresholds as in previous work [55] for several reasons: 1) this is not an established practice in the periodontal literature linking clinical and serological evidence of disease, 2) some of the antibodies included in the analyses are associated with periodontal health, and 3) the sample size was insufficient for such analyses. Some information could potentially be lost when grouping individual subjects into antibody categories rather than analyzing antibody values as continuous variables. However, the antibody level values were not normally distributed, nor could a normal distribution be achieved through conventional transformations (i.e., log-based or quadratic) given that a substantial number of individuals had no detectable levels for several antibodies, particularly *A. naeslundii*.

The possibility of a small-sample bias is also recognized. However, we used previously described methodology [23], [24], [31] to limit this bias, and the case-cohort sample reasonably approximated the characteristics of the overall study cohort. For example, in the present sample, older age and lower educational attainment conferred risk for incident AD in a similar manner as in the overall WHICAP cohort (data not shown). Although the associations of single antibody analyses were less precise, the findings are commensurate with the Cox-proportional hazards regression models. Importantly, simultaneous inclusion of both *A. naeslundii* and *E. nodatum* in the model resulted in nearly identical findings using both statistical methods. Moreover, the prevalence of high antibody levels was similar to the prevalence of other risk factors for dementia including DM [56] and stroke. [57] Given the small sample size of this study, adjustment for inflation of Type 1 error was not feasible and remains a limitation, given that several antibodies were jointly entered in the regression models. Furthermore, it is well established that periodontitis is not a mono-infection but rather a polybacterial disruption of host homeostasis. Consequently, antibody levels to several periodontal microbiota are interrelated, as also demonstrated by the correlation matrix in **Table S1**. Finally, persons with baseline mild cognitive impairment were not excluded and thus the possibility of reverse causation, i.e., that cognitive impairment may have affected the antibody levels to periodontal microbiota, cannot be ruled out.

The levels of serum antibodies to periodontal bacteria alone do not capture the complex interplay between bacterial infection and host response in the context of periodontitis, and as suggested here, possibly AD. The present findings are based on a limited sample, and their external validity needs to be confirmed in independent cohorts. Notably, since the development of this study, serum antibodies to other periodontal organisms not included in the panel investigated here have also been identified as potential risk markers for AD and mild cognitive impairment. [58] As recently reviewed elsewhere, [17] further multimodal exploration of the role of periodontal infections, other oral health markers, and the systemic host response may elucidate a novel potential causal pathway for cognitive impairment among the elderly.

## Supporting Information

S1 Figure Cox-proportional hazards regression of dementia-free survival of the association with high *A. naeslundii* antibody levels above a clinically defined threshold [9] (solid line) and those with low *A. naeslundii* antibody levels (dotted line) in a fully adjusted model (controlling for age at phlebotomy, APOE status, gender, education, hypertension, smoking, stroke, and diabetes).
(DOCX)

S2 Figure Cox-proportional hazards regression of dementia-free survival of the association with high *E. nodatum* antibody levels above a clinically defined threshold [9] (solid line) and those with low *E. nodatum* antibody levels (dotted line) in a fully adjusted model (controlling for age at phlebotomy, APOE status, gender, education, hypertension, smoking, stroke, and diabetes).
(DOCX)

S1 Table Aa: *A. actinomycetemcomitans*, Pg: *P. gingivalis*, Tf: *T. forsythia*, Td: *T. denticola*, Cr: *C. rectus*, En: *E. nodatum*, and An: *A. naeslundii*.
(DOCX)

S1 Data This dataset provides the periodontal antibody levels (ng/ml) for all 219 participants included in this study.
(XLSX)
