# Supplementary figures and images for: Serum IgG Antibody Levels to Periodontal Microbiota Are Associated with Incident Alzheimer Disease
Source: PLoS One. 2014 Dec 18;9(12):e114959. doi: 10.1371/journal.pone.0114959 (PMC4270775; doi:10.1371/journal.pone.0114959)

**Figure S1**


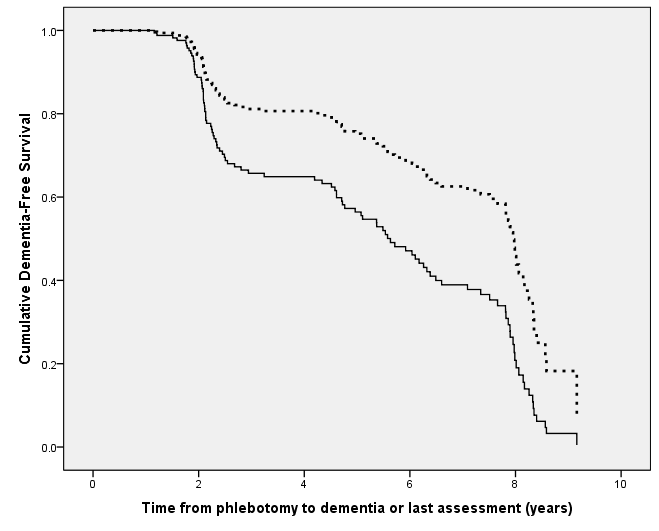

Supplement: S1 Figure — Cox-proportional hazards regression of dementia-free survival of the association with high A. naeslundii antibody levels above a clinically defined threshold [9] (solid line) and those with low A. naeslundii antibody levels (dotted line) in a fully adjusted model (controlling for age at phlebotomy, APOE status, gender, education, hypertension, smoking, stroke, and diabetes). (DOCX) [file pone.0114959.s001.docx]

**Figure S2**


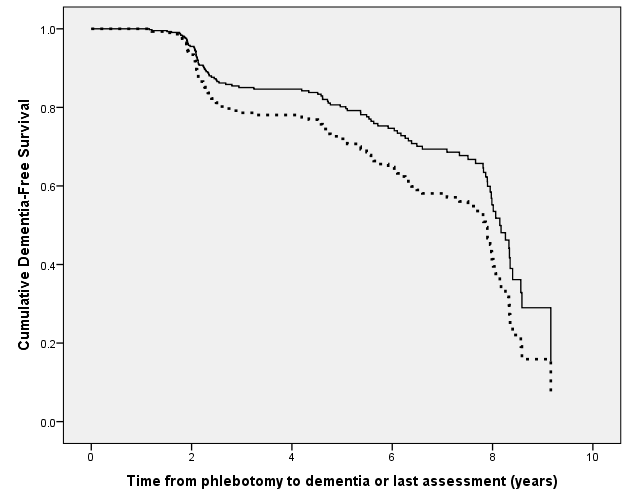

Supplement: S2 Figure — Cox-proportional hazards regression of dementia-free survival of the association with high E. nodatum antibody levels above a clinically defined threshold [9] (solid line) and those with low E. nodatum antibody levels (dotted line) in a fully adjusted model (controlling for age at phlebotomy, APOE status, gender, education, hypertension, smoking, stroke, and diabetes). (DOCX) [file pone.0114959.s002.docx]
