# Supplementary material for: Serum IgG Antibody Levels to Periodontal Microbiota Are Associated with Incident Alzheimer Disease
Source: PLoS One. 2014 Dec 18;9(12):e114959. doi: 10.1371/journal.pone.0114959 (PMC4270775; doi:10.1371/journal.pone.0114959)
Supplement: S1 Table — Aa: A. actinomycetemcomitans, Pg: P. gingivalis, Tf: T. forsythia, Td: T. denticola, Cr: C. rectus, En: E. nodatum, and An: A. naeslundii. (DOCX) [file pone.0114959.s003.docx]

|  | | Aa | Pg | Tf | Td | Cr | En | An |
| --- | --- | --- | --- | --- | --- | --- | --- | --- |
| Aa | *r* |  | 0.09 | 0.19 | 0.19 | 0.12 | 0.12 | 0.12 |
|  | *p-value* |  | 0.18 | 0.01 | 0.01 | 0.07 | 0.07 | 0.08 |
|  |  |  |  |  |  |  |  |  |
| Pg | *r* | .09 |  | 0.13 | 0.24 | 0.40 | .05 | .25 |
|  | *p-value* | .18 |  | 0.06 | <0.001 | <0.001 | .437 | <0.001 |
|  |  |  |  |  |  |  |  |  |
| Tf | *r* | 0.19 | 0.13 |  | 0.60 | 0.23 | 0.37 | .22 |
|  | *p-value* | 0.01 | .064 |  | .000 | .001 | .000 | .001 |
|  |  |  |  |  |  |  |  |  |
| Td | *r* | 0.19 | 0.24 | 0.60 |  | 0.29 | 0.50 | 0.31 |
|  | *p-value* | 0.01 | <0.001 | <0.001 |  | <0.001 | <0.001 | <0.001 |
|  |  |  |  |  |  |  |  |  |
| Cr | *r* | 0.12 | 040 | 0.23 | 0.29 |  | 0.15 | 0.17 |
|  | *p-value* | 0.07 | <0.001 | 0.001 | <0.001 |  | 0.03 | 0.01 |
|  |  |  |  |  |  |  |  |  |
| En | *r* | 0.12 | 0.05 | 0.37 | 0.50 | 0.15 |  | 0.36 |
|  | *p-value* | 0.07 | 0.44 | <0.001 | <0.001 | 0.031 |  | <0.001 |
|  |  |  |  |  |  |  |  |  |
| An | *r* | 0.12 | 0.25 | 0.22 | 0.31 | 0.17 | 0.36 |  |
|  | *p-value* | 0.08 | <0.001 | 0.001 | <0.001 | 0.01 | <0.001 |  |

**Table S1: Correlation matrix of high levels**[**^9^**](#_ENREF_9) **of serum IgG antibody to periodontal microbiota**
